# Recovery from social isolation requires dopamine in males, but not the autism-related gene *nlg3* in either sex

**DOI:** 10.1098/rsos.240604

**Published:** 2024-07-31

**Authors:** Ryley T. Yost, Andrew M. Scott, Judy M. Kurbaj, Brendan Walshe-Roussel, Reuven Dukas, Anne F. Simon

**Affiliations:** ^1^ Department of Biology, Western University, London, Ontario, Canada; ^2^ St Joseph’s Healthcare, Hamilton, Ontario, Canada; ^3^ Department of Psychology, Neuroscience and Behaviour, Animal Behaviour Group, McMaster University, Hamilton, Ontario, Canada

**Keywords:** social behaviour, social isolation, social space, neuroligin, dopamine, recovery

## Abstract

Social isolation causes profound changes in social behaviour in a variety of species. However, the genetic and molecular mechanisms modulating behavioural responses to social isolation and social recovery remain to be elucidated. Here, we quantified the behavioural response of vinegar flies to social isolation using two distinct protocols (social space preference and sociability, the spontaneous tendencies to form groups). We found that social isolation increased social space and reduced sociability. These effects of social isolation were reversible and could be reduced after 3 days of group housing. Flies with a loss of function of *neuroligin3* (orthologue of autism-related *neuroligin* genes) with known increased social space in a socially enriched environment were still able to recover from social isolation. We also show that dopamine (DA) is needed for a response to social isolation and recovery in males but not in females. Furthermore, only in males, DA levels are reduced after isolation and are not recovered after group housing. Finally, in socially enriched flies mutant for *neuroligin3*, DA levels are reduced in males, but not in females. We propose a model to explain how DA and *neuroligin3* are involved in the behavioural response to social isolation and its recovery in a dynamic and sex-specific manner.

## 1. Introduction

The environment an individual has been exposed to influence social behaviour by affecting the interactions between individuals [1]. Social isolation, or the absence of interactions with others, has profound effects on mental and physical health in humans [2–4]. During the COVID-19 times, forced social isolation had negative consequences on families and individuals [5]. Higher risk individuals such as children and people with neuropsychiatric disorders, like autism spectrum disorders, face more challenges to their physical and mental health because of changes to their behavioural and environment support [6,7]. It is important to understand the interactions between genetic predispositions and altered social environment, as well as the possibility to recover from social isolation.

Social isolation causes changes to the social behaviour in several organisms, including a variety of behavioural deficits in monkeys [8–11]; deficits in social interactions, aggression, fear and anxiety in mice [12–18] and decreased social affiliation in bees [19]. In *Drosophila melanogaster*, social isolation leads to physiological and behavioural changes, as described in recent reviews [20–22]. For example, lifespan appears to depend on the social context in a complex manner, as social isolation has been reported to decrease [23] or increase [24] lifespan, possibly depending on the number of flies in the social environment, strains and diet. Additional changes include decreased fibre number in the mushroom bodies [25], changes to neural excitability [26], chemical communication [27,28], olfactory memory [29], courtship and courtship memory [30], sleep patterns [31,32], locomotion [33], circadian rhythm [30], aggression [26,34–36], sociability [37] and social network structure [38,39]. Isolated male flies are much more territorial and defend food patches and access to mates more than socially enriched flies [40]. Our work and others’ have identified that isolated flies have increased social space [41–43], a measure of inter-individual distances.

Plastic changes in behaviour are important for an organism to adapt to a changing environment. Changes in social behaviour because of isolation can be partially or fully recovered in monkeys [10,11], mice [13,14,44] and bees [19]. Flies are no exception. In *Drosophila*, sleep and aggression levels were recovered after group housing [31,45]. However, more research needs to be conducted on the recovery from isolation of other *Drosophila* social behaviours.

In addition to social isolation, social space is modulated by social experience [41]; physiological state such as hunger [46], age of the individual and age of the parents [46,47]; time of the day [46,48]; viral infections, as well as exposure to environmental toxins [49–53] or anti-oxidant [49]. Orthologues of several candidate genes for neurodevelopmental disorders in humans also influence social space in *Drosophila*, as reported for example, not exhaustively, in [54–61]. Finally, a number of sensory modalities, neurocircuitry and synaptic proteins underlying social spacing have been identified [42,43,46–49,54,61–68]. One identified synaptic protein is *neuroligin3*, a post-synaptic cell adhesion molecule that regulates synaptic development and function and is an orthologue to a human autism candidate gene [42,69,70]. We have previously shown *nlg3* to be important for social space [42] and sociability (the tendency to engage in non-aggressive interactions with conspecifics [37]). We also showed that *nlg3* is required for a typical response to social isolation, but that the NLG3 protein levels are unchanged after social isolation [42]. However, the role of *nlg3* in recovery from social isolation has not been studied.

Another important molecule for social behaviour in the fly is dopamine (DA), a monoamine neurotransmitter [71–73]. We and others have previously shown that DA modulates social space [48] and is important for a response to social isolation. Specific dopaminergic neural circuity is in part responsible for the behavioural modification after isolation [43], and DA levels in males are reduced after isolation [31]. This could be because the levels of the transcripts of one of the DA biosynthesis enzymes (dopa decarboxylase) have been found to be reduced in isolated male flies’ purified dopaminergic neurons. In fact, many transcriptional and epigenetic changes occur in dopaminergic neurons in response to isolation [74]. However, the role of DA in social recovery has not been identified. In addition, evidence suggests that *nlg3* and DA are part of a similar neurocircuitry or involved in a similar pathway responsible for behaviour regulation [75–77]; however, their dual involvement in the regulation of social space needs more attention.

In this study, we examined the effect of social isolation on two measures of social behaviour: social space [41] and sociability [37]. We further investigated the role of *nlg3* and DA in the recovery from social isolation. Finally, we propose a model for the integrated regulation of *nlg3* and DA in the behavioural response to the social environment.

## 2. Methods

### 2.1. Fly stocks and husbandry

All fly lines were maintained in mixed-sex groups in bottles on JazzMix media or our own food made following the same recipe (brown sugar, corn meal, yeast, agar, benzoic acid, methyl paraben and propionic acid; Fisher Scientific, Whitby, Ontario, Canada) at 25°C, 50% relative humidity on a 12 L : 12 D cycle. All flies reared in bottles for experimental use were a maximum of 14 days old to avoid variation in behaviour resulting from older parents [47]. Fly lines were obtained from the following places: CS was obtained from the laboratory of Seymour Benzer in 1998, *nlg3^Def1^* are from Dr Brian Mozer [42], *w;; TH-GAL4* was provided by Dr Serge Birman and RNAi against tyrosine hydroxylase (TH) (*w;; UAS-THmiR-G*) was a gift from Dr Mark Wu [43]. All lines except *w;; UAS-THmiR-G* were outcrossed five times to our control line, CS, to reduce variation in behaviour caused by genetic background. Crosses used to generate *TH>THmiR*-G and their appropriate genetic controls can be found in the electronic supplementary material, figure S1.

### 2.2. Generation of isolation and recovery treatments

**Figure 1.**
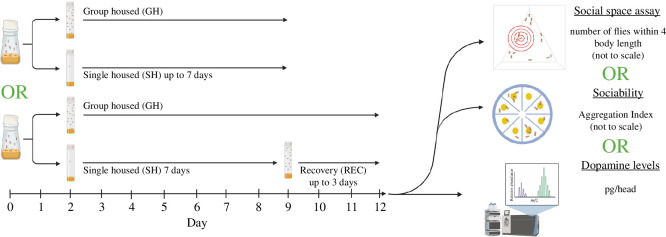
Schematic of the experimental paradigm and parameters measured in this study. Flies were group housed in mixed-sex bottles for 2 days after eclosing to adulthood, prior to being single housed or remained group housed. Flies remained single or group housed for up to 7 days (reaching the age of 9-days-old). The length of isolation is always 7 days unless indicated in the figure. After 7 days of being group or single housed, flies were tested in either the social space assay, sociability assay or used to determine DA levels. Separate populations of flies followed the same experimental design but were group housed for up to 3 days (to test recovery, reaching the age of 12-days-old) before behaviour or molecular experiments took place. All flies in each independent repeat were naive to the tests performed, and no flies were used in multiple behavioural or molecular experiments. Created using BioRender.

Mated flies used in all experiments were collected from bottles at 1 day old and remained mixed-sex in new bottles for one more day (2 days total) to allow mating (see confirmation of mating status below) to avoid the effects of mating status on social space [41]. Following, socially isolated flies were transferred to individual vials using cold anaesthesia and remained single housed for 2, 4 or 7 days. Group-housed age-matched control flies were kept in mixed-sex bottles for the same duration as the isolated flies, such that the group-housed control flies for isolation were 4, 6 and 9-days-old, respectively. To test for a recovery after social isolation, flies isolated for 7 days were transferred to mixed-sex bottles for either 2 or 3 days. Group-housed flies used as a control for the recovery treatment were maintained in mixed-sex bottles until tested with the recovery flies, such that the group-housed control flies for isolation then recovery were 11- and 13-days-old, respectively. To test the effect of group housing density on social space, we sorted the group-housed flies into vials mixed-sex containing 2, 6 or 16 flies and a separate uncontrolled amount (random) in a bottle. The experimental design for our experiments comparing group-housed and isolated or recovered flies can be found in figure 1.

To test for the effects of virginity on social isolation, pupae were collected from bottles and placed individually into vials and allowed to eclose over 24 h. Isolated virgin flies remained in individual vials, whereas group-housed virgin flies were transferred into vials containing 15 same-sex flies to maintain virginity.

#### 2.2.1. Females mating status at 2- and 4-days-old

One-day-old female flies were collected and maintained with males in bottles either 1 day (24 h) or 3 days. Females were then sexed under cold anaesthesia, and each female was placed alone in a fresh vial. Females were scored as having been mated when the third instar larvae were observed.

#### 2.2.2. Males mating status at 2- and 4-days-old

Single, 1-day-old male flies, sexed under cold anaesthesia, were placed with one 4-day-old virgin female in vials (based on the females results, more than 90% of females that age can mate). After either 1 day (24 h) or 3 days, the males were removed, and females remained in isolation until the third instar larvae were counted. For both sexes, vials with dead flies were not counted. We cannot ensure all flies have mated, even in the group housing treatment, so all flies exposed to the initial group housing are used in the behaviour experiments, including the small percentage of non-mated flies.

### 2.3. Social behaviour assays

Sociability tests were performed at McMaster University. All other experiments took place at Western University under similar settings.

#### 2.3.1. Fly handling prior to behaviour

Twenty-four hours prior to all behaviour assays, 15–17 flies that were group housed (either lifelong or after a few days of recovery) were collected using cold anaesthesia and placed in vials. Single-housed flies were kept isolated until right before testing. The morning of the experiment, all flies were transferred to new vials and allowed to habituate to the testing conditions of 25°C and 50% relative humidity for at least 2 h. The social space assay was conducted under uniform light in the same room between 13.00 and 17.00 (ZT 4–8) to decrease behavioural variation linked to diel periodicity. Finally, we used an internal control for genetic background in each experiment, as the entire dataset display differences in performances, depending on variables that we cannot control (refer, e.g. figure 1 and Yost *et al*. [42]).

#### 2.3.2. The social space assay

The social space assay was performed as previously described [41,42,66]. In short, once flies have settled at their preferred inter-individual distance, a photo was taken (after 20–50 min, depending on their genotype). Using the open-access software ImageJ (RRID:SCR_003070; [78]), the number of flies within four body lengths (approx. 1 cm) was determined for each fly in the chamber. The number of flies within four body lengths was averaged using all flies in the chamber, representing one individual replicate. This metric has been used in the previous studies [42,43]. The routines for image analysis are publicly available [42]. All datasets are the combination of 1–3 replicates per day and 3–5 independent days of testing. Each independent day was separated by at least a week to control for environmental factors beyond our control.

#### 2.3.3. Sociability assay

The sociability chamber consisted of a circular arena (90 mm wide by 20 mm high) divided into eight compartments with a hole in the centre to allow flies to enter any compartment. We added to each compartment a patch of fresh food coated with a layer of grapefruit–yeast suspension (3 g yeast in 100 ml grapefruit juice) to enhance attractiveness. We modified the chamber and performance of the assay from Scott *et al.* [37]. We transferred by mouth aspiration 16 same-sex flies to the chamber through a hole in the lid and allowed them to acclimate for 1 h. Experimenters blind to treatment then counted the number of flies in each chamber and calculated an aggregation index (sample variance divided by the mean number of flies in each chamber). The variance could take values between 0 and 32. For example, the least sociable option would have eight chambers of two flies each, with a variance of 0 and the corresponding aggregation index of 0. The most sociable situation would have seven chambers of 0, and one chamber with all 16 flies. This would have a variance of 32 and therefore an aggregation index of 16 (32/2). We tested flies in sessions of four replicate arenas per treatment over three consecutive weeks, for 12 arenas total per treatment (total *n* = 96).

### 2.4. Dopamine quantification

Adults were separated by sex, and DA extracted from their heads using the following procedure. Extraction occurred by flash freezing flies in liquid nitrogen followed by manual decapitation and homogenization of heads in 5 mM of ammonium acetate in 90% acetonitrile using microtissue grinders (Kimble Chase, USA). The supernatant was transferred and filtered through a 0.65 µm filter (Millipore) at 4°C, and samples were stored at −80°C before detection using liquid chromatography/mass spectrometry (LC/MS).

LC/MS analysis was performed using an Agilent 1260 Infinity LC system coupled to an Agilent 6230 Time of Flight (TOF) system. A XBridge C-18 column Rapid Resolution HT was used (4.6 Å approx. 150 mm, 3.5 μm, 600 bar, waters) at 25°C, and samples were eluted with a gradient of CH_3_CN (solvent B: 90% CH_3_CN in H_2_O, containing 0.1% formic acid) in H_2_O (solvent A: containing 0.1% formic acid). The UV lamp was set at 282 nm, and the injection volume was 10 µl. The flow rate was set to 0.4 ml min^−1^ and infused into an Agilent 6230 TOF-MS through a dual-spray electro ionization source with a gas temperature of 325°C flowing at 8 l min^−1^ and a nebulizer pressure of 35 psi. The fragmentor voltage was set to 175 V with a capillary voltage of 3500 V and a skimmer voltage of 65 V. The instrument was set in positive ESI mode, and quantification occurred using a standard curve of known DA concentrations (electronic supplementary material, figure S2). Total ion count was extracted using the Agilent MassHunter qualitative analysis software (v. B.05.00).

### 2.5. Statistics

Data were stored in Excel files and statistically analysed using GraphPad Prism (RRID:SCR_002798, v. 7.0 a for Mac, GraphPad software, La Jolla, California, USA, www. graphpad.com). We first assessed the normality and homoscedasticity of the data distribution prior to applying the appropriate parametric tests (simple *t*‐test or ANOVA or Welch *t*‐test/ANOVA when the standard deviations were different among groups). When performing statistical analysis of an experiment with only two groups, we used a *t*‐test. When there were more than two groups but only one variable, we used a one-way ANOVA. When we compared two variables across two or more groups, we used two-way ANOVAs. One-way and two-way ANOVAs were followed by Tukey’s or Sidak’s post hoc test (respectively) to identify significantly different points, while correcting for multiple comparisons. We used the traditional alpha level of 0.05 for all statistical tests [79]. On the graphs, we present the *p*-values for tests, and post hoc tests are performed only when significant. The results of all the comparisons, significant or not, can be found in tables 1–3.

**Table 1.** ANOVA table for all two-way ANOVAs performed. (Statistically significant results are in bold.)

experiment	figure number	effect	d.f.	*F*	*p*	Sidak post hoc		
social space						**comparison group**	**compared with**	***p***
days of isolation—males	2*a*	days	(2,46)	2.412	0.1009	group housed vs single housed	2 days	0.0529
		**isolation**	**(1,46)**	**22.01**	**<0.0001**		**4 days**	**0.0424**
		days * isolation	(2,46)	0.1534	0.8582		**7 days**	**0.0093**
days of isolation—females	**2** ***b***	**days**	**(2,47)**	**3.683**	**0.0327**	group housed vs single housed	2 days	0.9992
		**isolation**	**(1,47)**	**6.653**	**0.0131**		4 days	0.1404
		days * isolation	(2,47)	1.453	0.2441		7 days	0.0746
mating status—males	**3** ***a***	**isolation**	**(1,52)**	**35.69**	**0.0327**	group housed vs single housed	**mated**	**<0.0001**
		**mating status**	**(1,52)**	**4.223**	**0.0131**		**virgin**	**0.0007**
		isolation * mating status	(1,52)	0.2036	0.2441			
mating status—females	3*b*	**isolation**	**(1,52)**	**9.924**	**0.0027**	group housed vs single housed	**mated**	**0.0033**
		**mating status**	**(1,52)**	**5.269**	**0.0258**		virgin	0.4308
		isolation * mating status	(1,52)	2.131	0.1504			
Cs & *nlg3* ^*Def1*^ isolated—males	4*a*	**genotype**	**(1,32)**	**10.1**	**0.0033**	group housed vs single housed	**Cs**	**0.0001**
		**isolation**	**(1,32)**	**46.48**	**<0.0001**		***nlg3*** ^***Def1***^	**<0.0001**
		genotype * isolation	(1,32)	0.0086	0.9267			
Cs & *nlg3* ^*Def1*^ recovery—males	4*b*	**genotype**	**(1,37)**	**39.94**	**0.0002**	group housed vs recovery	Cs	0.9213
		**recovery**	**(1,37)**	**17.72**	**<0.0001**		***nlg3*** ^***Def1***^	**<0.0001**
		genotype * recovery	**(1,37)**	**13.62**	**0.0007**			
Cs & *nlg3* ^*Def1*^ isolated—females	4*c*	**genotype**	**(1,32)**	**13.71**	**0.0008**	group housed vs single housed	**Cs**	**<0.0001**
		**isolation**	**(1,32)**	**27.97**	**<0.0001**		*nlg3* ^*Def1*^	0.0769
		genotype * isolation	**(1,32)**	**5.37**	**0.027**			
Cs & *nlg3* ^*Def1*^ recovery—females	4*d*	**genotype**	**(1,34)**	**6.178**	**0.018**	group housed vs recovery	Cs	0.1326
		recovery	(1,34)	0.3197	0.5755		***nlg3*** ^***Def1***^	**0.0197**
		genotype * recovery	**(1,34)**	**10.6**	**0.0026**			
TH-miR isolation—males	5*a*	genotype	(2,35)	1.633	0.2099	group housed vs single housed	*th-gal4*/+	0.9851
		**isolation**	**(1,35)**	**4.631**	**0.0384**		***UAS-th-miR-G*** **/+**	**0.0003**
		genotype * isolation	**(2,35)**	**8.635**	**0.0009**		*th>th-miR-G*	0.5283
TH-miR recovery—males	5*b*	**genotype**	**(2,37)**	**9.018**	**0.0006**	group housed vs recovery	*th-gal4/+*	>0.9999
		recovery	(1,37)	4.631	0.7555		*UAS-th-miR-G/+*	0.999
		genotype * recovery	(2,37)	8.635	0.8263		*th>th-miR-G*	0.8707
TH-miR isolation—females	5*c*	**genotype**	**(2,37)**	**4.009**	**0.0265**	**group housed vs single housed**	***th-gal4/+***	**0.0188**
		**isolation**	**(1,37)**	**21.91**	**<0.0001**		***UAS-th-miR-G/+***	**0.0025**
		genotype * isolation	(2,37)	0.8892	0.4196		*th>th-miR-G*	0.2928
TH-miR recovery—females	5*d*	**genotype**	**(2,35)**	**5.124**	**0.0112**	group housed vs recovery	*th-gal4/+*	0.8403
		recovery	(1,35)	2.562	0.1184		*UAS-th-miR-G/+*	0.0667
		genotype * recovery	(2,35)	2.036	0.1457		*th>th-miR-G*	0.9661
**dopamine levels**						**comparison group**	**compared with**	***p***
Cs	5*e*	sex	(1,27)	1.665	0.2078	males grouped housed (GH) – 9 days old (9do)	***males SH 9do***	**0.0305**
		**social experience**	**(3,27)**	**3.043**	**0.0459**		*males GH 12do*	0.2372
		sex * social experience	(3,27)	1.13	0.3545		*males rec 12do*	>0.999
						females grouped housed (GH) – 9 days old (9do)	*females SH 9do*	0.9775
							*females GH 12do*	0.996
							*females REC 12 do*	0.5151
Cs and *nlg3* ^*Def1*^	6*a*	**sex**	**(1,7)**	**6.107**	**0.0428**	Cs males	*Cs females*	0.1531
		**genotype**	**(1,7)**	**17.21**	**0.0043**		***males nlg3*** ^***Def1***^	**0.0347**
		sex * genotype	(1,7)	2.797	0.1384		***females nlg3*** ^***Def1***^	**0.0179**
						Cs females	*males nlg3* ^*Def1*^	0.8203
							*females nlg3* ^*Def*^	0.4884
						*nlg3* ^*Def*^ males	*females nlg3* ^*Def*^	0.9935
*nlg3* ^*Def1*^	6*b*	sex	(1,16)	0.007	0.999	males grouped housed (GH) – 9 days old (9do)	*males SH 9do*	0.9922
		social experience	(3,16)	1.354	0.2922		*males GH 12do*	0.7168
		sex * social experience	(3,16)	0.921	0.3513		*males rec 12 do*	0.9969
							*females SH 9do*	0.9798
							*females GH 12do*	0.6796
							*females rec 12 do*	0.9989

**Table 2.** All Welch’s *t*-tests were performed. (Two-tailed when no hypothesis for direction and two-tailed when directions of change are hypothesized. Statistically significant results are in bold.)

experiment	figure number	comparison	one or two tail?	d.f.	*t*	*p*
sociability isolation—males	2*c*	group-housed versus single-housed males	two tailed	**12.08**	**4.039**	**0.0016**
sociability isolation—females	2*c*	group-housed versus single-housed females	two tailed	**13.37**	**2.578**	**0.0225**
social space recovery 2 day—males	2*d*	group-housed versus recovery males	two tailed	14.46	0.4815	0.6373
social space recovery 2 day—females	2*d*	group-housed versus recovery females	two tailed	**11.3**	**3.673**	**0.0035**
social space recovery 3 day—males	2*e*	group-housed versus recovery males	one tailed	9.545	1.542	0.0778
social space recovery 3 day—females	2*e*	group-housed versus recovery females	one tailed	12.67	1.465	0.1672
sociability recovery 3 day—males	2*f*	group-housed versus recovery males	one tailed	21.96	0.3738	0.7121
sociability recovery 3 day—females	2*f*	group-housed versus recovery females	one tailed	15.01	1.954	0.0696

**Table 3.** ANOVA table for all one-way ANOVAs performed. (Statistically significant results are in bold.)

experiment	figure number	d.f.	*F*	*p*	Sidak post hoc		
					**comparison group**	**compared with**	***p***
group-housed density—males	2*c*	**(4,40)**	**30.87**	**<0.0001**	single housed	**2**	**<0.0001**
						**6**	**<0.0001**
						**16**	**<0.0001**
						random	**<0.0001**
group-housed density—females	2*c*	**(4,40)**	**31.35**	**<0.0001**	single housed	**2**	**<0.0001**
						**6**	**<0.0001**
						**16**	**<0.0001**
						random	**<0.0001**

## 3. Results

### 3.1. Isolation for 7 days leads to increased social space and decreased sociability

Seven days of social isolation leads to an increased social space [41]. We wanted to determine whether we could reduce the duration of social isolation and still influence social space.

Because mating status influences social space and virgin flies are further apart than mated flies [41], we needed to first assess how long flies should be group-housed post-emergence to avoid the confounding effect of virginity on social space. We found that 101 out of 112 (90.2%) of females were mated and 55 out of 72 (76.4%) of males were able to mate after spending 4–5 days, mixed with females (electronic supplementary material, table S1). In comparison, 94 out of 103 (91.2%) of females were mated 2 days after adult emergence spent with males, and 69 out of 81 (85.2%) of males of the same age were able to mate. The ages at which the flies were tested had no significant effect on their mating capabilities (*χ*²_₁_ = 0.022, *p* = 0.88 for females and *χ*²_₁_ = 1.92, *p* = 0.166 for males). We thus decided to expose our flies to 2 days of group housing post-emergence, before socially isolating them for 2, 4 or 7 days, as we now know that the same percentage of flies have mated by that age, as when they are 4–5 days. We then assessed their social space by recording the number of flies present within four body lengths.

**Figure 2.**
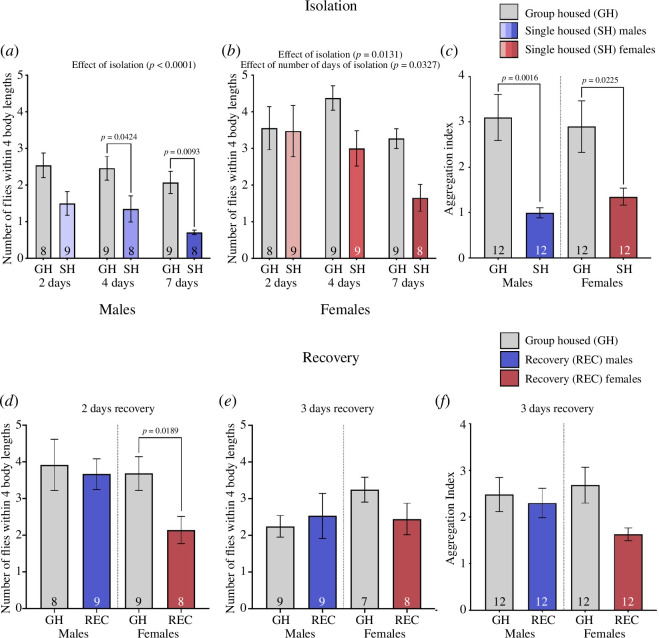
Isolation for 7 days leads to increased social space and decreased sociability, but flies recover after 3 days of group housing. (*a,b,d,e*) Social space presented in terms of mean number of flies within four body lengths ± s.e.m. (*c,f*) Effect of 2, 4 or 7 days of isolation in males (*a*) and females (*b*) social space. (*a*) Male flies had fewer flies within four body lengths after 2, 4 and 7 days of isolation (two-way ANOVA and Sidak post hoc test—*F*_1,46_ = 22.01, *p* < 0.0001). (*b*) Females had fewer flies within four body lengths after isolation and that number decreased further with increasing days of isolation (two-way ANOVA—effect of isolation: *F*_1,47_ = 6.653, *p* = 0.0131; effect of days of isolation: *F*_2,47_ = 3.687, *p* = 0.0367). (*c*) Aggregation index in males and females after 7 days of isolation. Both males (Welch’s *t*‐test: *t*_12.08_ = 4.039, *p* = 0.0016) and females (Welch’s *t*‐test: *t*_13.37_ = 2.578, *p* = 0.0225) had a lower aggregation index after 7 days of isolation. (*d,e*) Males and females after 2 (*d*) and 3 (*e*) days of group housing (recovery) following 7 days of isolation. (*d*) Males showed no difference in the number of flies within four body lengths after 2 days of group housing compared with males continuously group housed. However, females had fewer flies within four body lengths compared with females group housed continuously (Welch’s *t*‐test: *t*_15.13_ = 2.626, *p* = 0.0189). (*e*) Both males and females were not different in the number of flies within four body lengths comparing group-housed and recovery flies (one-way ANOVA: *F*_3,28_ = 0.9434, *p* = 0.4329). (*f*) Sociability presented as the mean aggregation index ± s.e.m. of males and females group housed and recovery. Males and females did not differ in aggregation index between group housed and flies isolated 7 days, with 3 days of recovery. *n* = 7–12 for all treatments. On the graph, the results of the post hoc tests are shown only when *p* < 0.05. The results of all the comparisons, significant or not, can be found in tables 1–3. Grey shade: control group housed (GH); coloured shades: treatments; blue shades: single housed (SH) and recovery from social isolation (REC) males; red shades: SH and REC females.

CS (our control line) males had fewer flies within four body lengths after social isolation compared with group-housed flies at all three time points (2, 4 or 7 days) (figure 2*a*; two-way ANOVA results in table 1; effect of isolation: *F*_1,46_ = 22.01, *p* < 0.0001). There was no effect of 2 days of social isolation in CS females. Only after 4 and 7 days of isolation did CS females have fewer flies within four body lengths, and the increase in social space was larger with increasing number of days flies isolated (figure 2*b*; two-way ANOVA results in table 1; effect of isolation: *F*_1,47_ = 6.653, *p* = 0.0131; effect of days of isolation: *F*_2,47_ = 3.683, *p* = 0.0327). The effect of isolation after 7 days was similar to the effects found in earlier studies [41]. Moving forward, we used 7 days of isolation for the rest of the experiments as 7 days had the largest increase in social space in males and females.

We next used another measure of social behaviour, sociability [37], to examine the effect of isolation. Both males and females had a lower aggregation index in isolated flies compared with those group housed (figure 2*c*; table 2; males—Welch’s *t*‐test: *t*_12.08_ = 4.039, *p* = 0.0016; females—Welch’s *t*‐test: *t*_13.37_ = 2.578, *p* = 0.0225).

### 3.2. Males and females recover from isolation after 3 days of group housing

As recovery from isolation has been noted in various organisms including *D. melanogaster* (§1), we tested whether social space and sociability could also be recovered in flies that were group housed following isolation. We started with 2 days of group housing following isolation and found that males were not different in flies within four body lengths indicating they had recovered in social space; however, females still had a lower number of flies within four body lengths in the recovery compared with group-housed flies (figure 2*d*; table 2; Welch’s *t*‐test: *t*_15.13_ = 2.626, *p* = 0.0189). Because females had not fully recovered after 2 days of group housing post isolation, we tested social space in flies that had 3 days of group housing after 7 days of isolation. Again, males were not different in flies within four body lengths; however, females were now also not different in flies within four body lengths in recovery and group-housed flies indicating that females also can recover from isolation but take longer than males (one more day in this case—figure 2*e*; table 3). A period of 3 days of group housing following 7 days of isolation was then used for all other experiments investigating recovery. Finally, we tested sociability in males and females after 3 days of recovery. Males and females did not differ in aggregation index in recovery and group-housed flies, indicating that both sexes recovered (figure 2*f*; table 3).

### 3.3. Mating status does not alter social space in response to isolation

**Figure 3.**
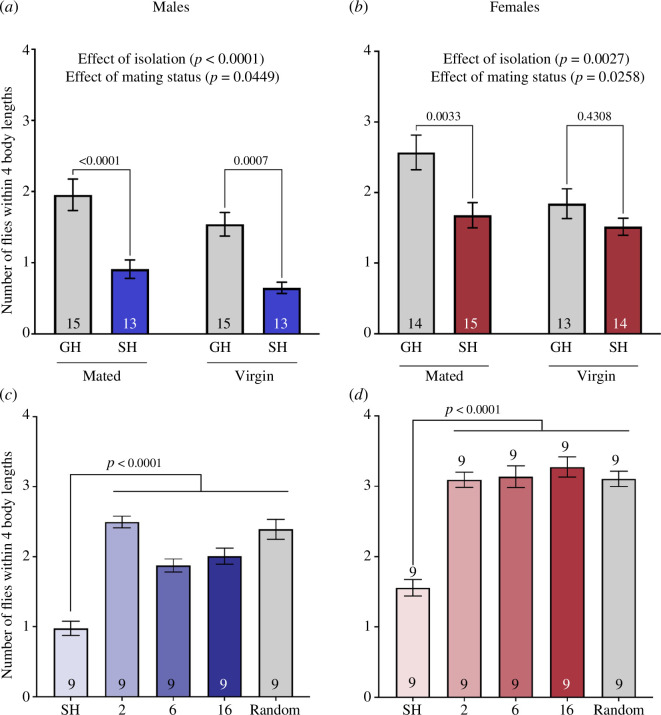
Mating status does not change social space in response to isolation and group housed with a single fly is enough to avoid a larger social space. (*a,b*) Number of flies within four body lengths in males (*a*) and females (*b*) group and single housed while mated or virgin. (*a*) Male flies had less flies within four body lengths after isolation (two-way ANOVA—effect of isolation: *F*_1,52_ = 34.69, *p* < 0.0001) regardless of mating status. Males had less flies within four body lengths when virgin compared with mated flies (two-way ANOVA—effect of mating status: *F*_1,52_ = 4.223, *p* = 0.0449). (*b*) Female flies had less flies within four body lengths after isolation (two-way ANOVA—effect of isolation: *F*_1,52_ = 9.924, *p* = 0.0027) regardless of mating status. Females had less flies within four body lengths when virgin compared with mated flies (two-way ANOVA—effect of mating status: *F*_1,52_ = 5.269, *p* = 0.0258). Grey shades: control group housed (GH); coloured shades: treatments; blue shades: single-housed (SH) males; red shades: SH females. (*c,d*) Number of flies within four body lengths in males (*c*) and females (*d*) reared single housed or group housed with 2, 6, 16 or a random number of flies. Single-housed flies had less flies within four body lengths than group-housed flies that were reared with any number of individuals in males ((*c*) one-way ANOVA with Holm-Sidak post hoc: *F_4_*_,40_ = 30.87, *p* < 0.0001) and females ((*d*) one-way ANOVA with Holm–Sidak post hoc: *F*_4,40_ = 31.35, *p* < 0.0001). Grey shades: random number of flies; coloured shades: varying number of flies, from 16 to one (SH: single housed); blue shades: males; red shades: females. On the graph, the results of the post hoc tests are shown only when *p* < 0.05. The results of all the comparisons, significant or not, can be found in tables 1–3. *n* = 9–13 for all treatments. Bars: mean ± s.e.m.

We have previously reported that virgin flies have an increased social space [41] as do isolated flies ([42] and figure 1); however, we have not tested virgin flies in response to isolation, so we next tested the effect of isolation on social space in mated and virgin flies. There were less flies within four body lengths after isolation in both mated and virgin males (figure 3*a*; two-way ANOVA results in table 1; effect of isolation: *F*_1,52_ *=* 34.69*, p <* 0.0001). In addition, virgin males had less flies within four body lengths when group and single housed compared with mated group and single housed (figure 3*a*; two-way ANOVA results in table 1; effect of mating status: *F*_1,52_ *=* 4.223*, p =* 0.0449). In females, we found that in mated flies, single-housed flies had less flies within four body lengths than group-housed individuals (figure 3*b*; two-way ANOVA results in table 1; effect of isolation: *F*_1,52_ *=* 9.924*, p =* 0.0027); however, group- and single-housed virgins had similar flies within four body lengths (*p* = 0.4308). Similar to males, virgin females had less flies within four body lengths when group and single housed compared with mated group and single housed figure 3*b*; two-way ANOVA results in table 1; effect of mating status: *F*_1,52_ *=* 5.269*, p =* 0.0258).

### 3.4. Group-housed density does not alter social space

We next tested what happens when group-housed flies are reared at different densities ranging from two flies up to an uncontrolled or random number of flies. We found that single-housed flies have less flies within four body lengths compared with group-housed flies at all densities in males (figure 3*c*; table 3; one-way ANOVA with Holm–Sidak post hoc: *F*_4,40_ = 30.87, *p* < 0.0001) and females (figure 3*d*; table 3; one-way ANOVA with Holm–Sidak post hoc: *F*_4,40_ = 31.35, *p* < 0.0001).

### 3.5. The lack of *nlg3* does not prevent from recovering from social isolation

**Figure 4.**
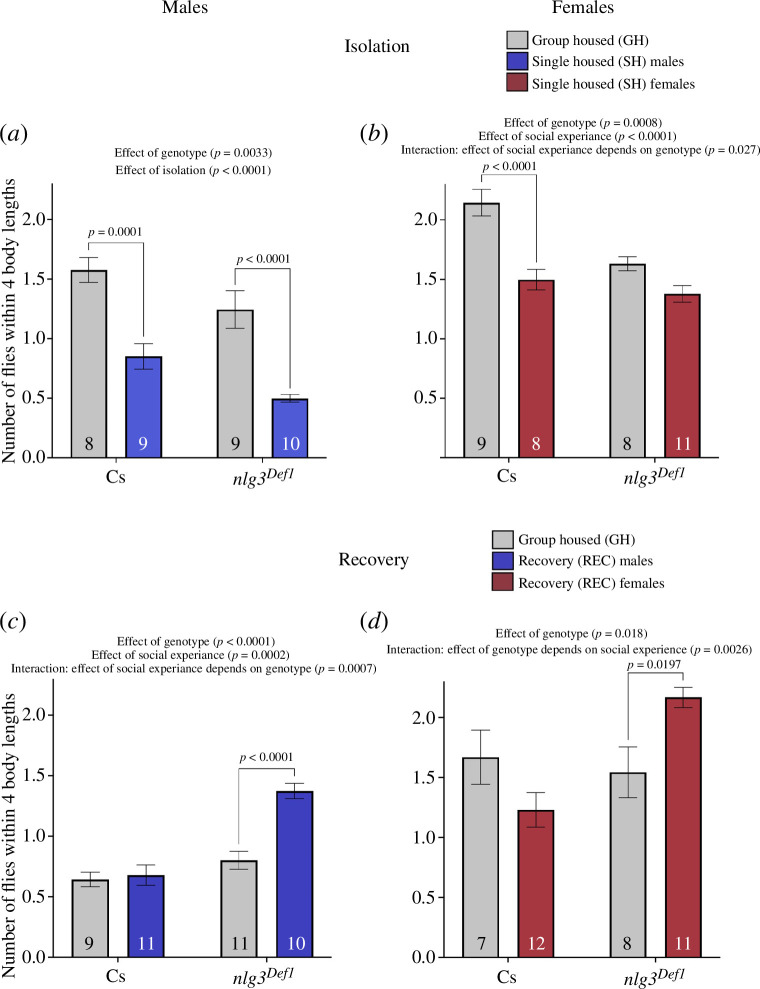
*nlg3^Def1^* flies have decreased social space after a recovery from isolation. (*a*, *b*) Number of flies within four body lengths in group- and single-housed males (*a*) and females (*b*) group housed. (*a*) Single-housed male flies had decreased flies within four body lengths compared with group-housed flies in both CS and *nlg3^Def1^* (two-way ANOVA—effect of social experience: *F*_1,32_ = 46.48, *p* < 0.0001), and *nlg3^Def1^* flies had less flies within four body lengths in both group-housed and single-housed (two-way ANOVA—effect of genotype: *F*_1,32_ = 10.10, *p* = 0.0033). (*b*) CS females had less flies within four body lengths in single-housed compared with group-housed flies; however, *nlg3^Def1^* flies were not different between group- and single-housed flies (two-way ANOVA—effect of social experience: *F*_1,32_ = 27.97, *p* < 0.0001; effect of genotype: *F*_1,32_ = 13.71, *p* = 0.0008; interaction of social experience and genotype: *F*_1,32_ = 5.370*, p* = 0.0270). Grey shades: control group housed (GH); coloured shades: treatments; blue shades: single housed (SH); males; red shades: SH females. (*c,d*) Number of flies within four body lengths in group and recovered males (*c*) and females (*d*). (*c*) The number of flies within four body lengths for CS males was not different in group housed versus recovery flies; however, *nlg3^Def1^* flies had an increased number of flies within four body lengths in recovery compared with group-housed flies (two-way ANOVA—effect of social experience: *F*_1,37_ = 17.72, *p* = 0.0002; effect of genotype: *F*_1,37_ = 34.94, *p* < 0.0001; interaction of social experience and genotype: *F_1,37_* = 13.62*, p* = 0.0007). (*d*) The number of flies within four body lengths for CS females was not different in group-housed versus recovery flies; however, *nlg3^Def1^* females had an increased number of flies within four body lengths in recovery compared with group-housed flies (two-way ANOVA—effect of social experience: *F*_1,34_ = 0.3197, *p* = 0.5755; effect of genotype: *F*_1,34_ = 6.178, *p* = 0.0180; interaction of social experience and genotype: *F*_1,34_ = 10.60*, p* = 0.0026). Grey shades: group housed (GH); coloured shades: recovery from social isolation (REC); blue shades: males; red shades: females. On the graph, the results of the post hoc tests are shown only when *p* < 0.05. The results of all the comparisons, significant or not, can be found in tables 1–3. *n* = 7–12 for all treatments. GH: group housed, SH: single housed, REC: recovery.

We have previously shown that *nlg3* is important for a typical social space response to isolation [42]. We wondered whether *nlg3* is also important for the recovery from social isolation. In males, both CS and *nlg3^Def1^* had less flies within four body lengths in single-housed compared with group housed-flies and *nlg3^Def1^* had less flies within four body lengths than CS (figure 4*a*; two-way ANOVA results in table 1; effect of social experience: *F*_1,32_ *=* 46.48*, p <* 0.0001; effect of genotype: *F*_1,32_ *=* 10.10*, p =* 0.0033). In females, CS had less flies within four body lengths when single housed; however, *nlg3^Def1^* females had no difference in the number of flies within four body lengths compared with group-housed flies (figure 4*b*; two-way ANOVA results in table 1; effect of social experience: *F*_1,32_ *=* 27.97*, p >* 0.0001; effect of genotype: *F*_1,32_ *=* 13.71*, p =* 0.0008; interaction of social experience and genotype: *F*_1,32_ *=* 5.370*, p =* 0.0270).

When testing the recovery, CS males did not differ in the number of flies within four body lengths; however, *nlg3^Def1^* males had increased flies within four body lengths in the recovery treatment compared with group-housed *nlg3^Def1^* flies (figure 4*c*; two-way ANOVA results in table 1; effect of social experience: *F*_1,37_ *=* 17.72*, p =* 0.0002; effect of genotype: *F*_1,37_ *=* 34.94*, p >* 0.0001; interaction of social experience and genotype: *F*_1,37_ *=* 13.62*, p =* 0.0007). In females, CS flies recovered from isolation did not have a different number of flies within four body lengths compared with flies always group housed; however, *nlg3^Def1^* flies had increased number of flies within four body lengths in the recovery treatment compared with group-housed flies (figure 4*d*; two-way ANOVA and Tukey post hoc results in table 1; effect of social experience: *F*_1,34_ *=* 0.3197*, p =* 0.5755; effect of genotype: *F*_1,34_ *=* 6.178*, p =* 0.0180; interaction of social experience and genotype: *F*_1,34_ *=* 10.60*, p =* 0.0026).

This recovery experiment is repeated in the electronic supplementary material, figure S3 (those data were in fact collected at the same time as the isolation treatments published by Yost *et al.* [42]). Combined, the data indicate that *nlg3* is not required for recovery to occur.

### 3.6. Dopamine is required for a response to isolation and decreases after isolation in a sex-specific manner

DA is important not only for social space in both sexes [48] but also for the response to social isolation in males [43]. We tested to see whether we could recapitulate these results after isolation and determine if DA was important for the recovery from isolation. We drove a RNAi against the gene encoding for *TH*, the rate-limiting enzyme for DA biosynthesis (*TH*-RNAi, specifically the *UAS-THmiR-G*) using a *TH-gal4* driver.

**Figure 5.**
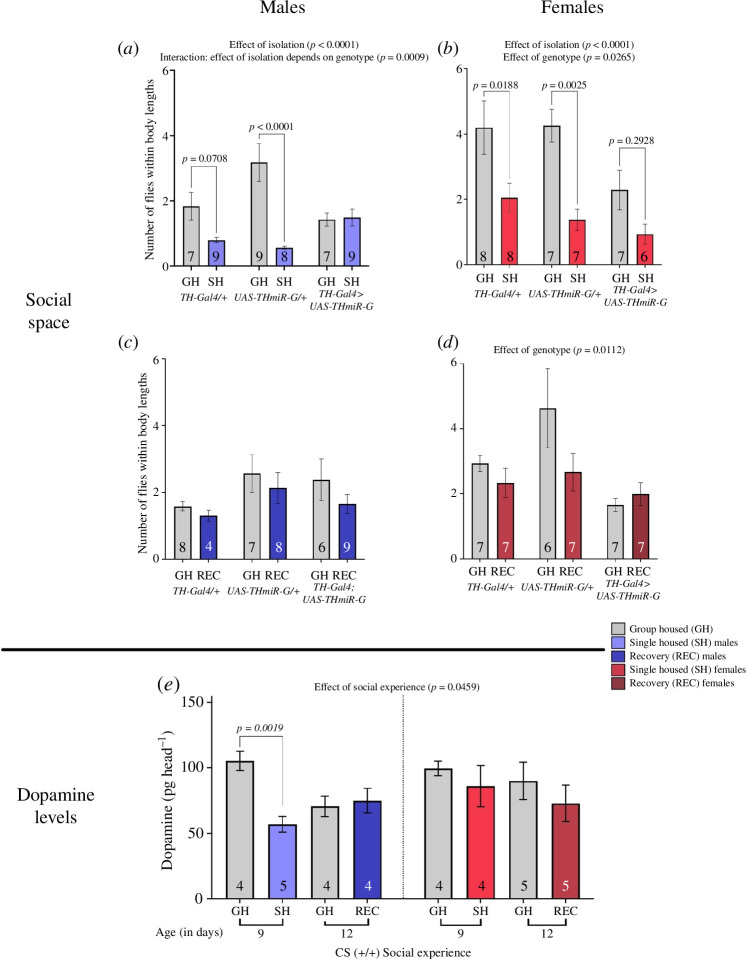
DA is important for a response to social isolation and decreases after isolation in males but not females. Social space measured in terms of number of flies within four body lengths. Bars: mean ± s.e.m. (*a,b*) Social space in isolated male (*a*) and female (*b*) flies. (*a*) The social space in male *TH>THmiR*-G was not different in isolated versus group-housed flies; however, *TH-GAL4/+* and *UAS-THmiR-G/+* males had lower flies within four body lengths when in isolation (two-way ANOVA—effect of isolation: *F*_1,43_ = 19.13, *p* < 0.0001; interaction between isolation and genotype: *F*_2,42_ = 8.254, *p* = 0.0009). (*b*) In females, all three genotypes had a decrease in the number of flies within four body lengths after isolation and *TH>THmiR* G flies had the lowest number of flies within four body lengths compared with their genetic controls (two-way ANOVA—effect of isolation: *F*_1,37_ = 21.91, *p* < 0.0001; effect of genotype: *F_2_*_,37_ = 4.009, *p* = 0.0265). (*c,d*) Social space in recovered male (*c*) and female (*d*) flies. (*c*) Male recovery flies in all genotypes were not different compared with group-housed males. (*d*) In females, all three genotypes were similar when comparing group-housed versus recovery treatments; however, *TH>THmiR-G* females had a less flies within four body lengths compared with the controls (two-way ANOVA—effect of genotype: *F*_2,35_ = 5.124, *p* = 0.0112). (*e*) DA levels (pg head^−1^) in males decreased in isolated compared with group-housed flies but do not differ in recovery flies compared with group housed in males or females (two-way ANOVA—effect of social experience: *F*_3,27_ = 3.043, *p* = 0.0459, but the only significant difference after correcting for multiple comparison through a Dunnett test is indicated on the graph, *p* = 0.0132). Appropriate age comparisons were made (refer §2 for details). *p < 0.05. ****p < 0.0001. Bars: mean ± s.e.m. On the graph, the results of the post hoc tests are shown only when *p* < 0.05. The results of all the comparisons, significant or not, can be found in tables 1–3. *n* = 5–9 for all treatments in social space. *n* = 4 for DA quantification. GH: group housed, light grey shade; SH: single housed, dark grey shade; REC: recovery from social isolation, medium grey shade.

In males, *TH>THmiR* G, there was no effect of social experience. We did observe a significant reduction in the number of flies within four body lengths with isolation in *TH-*Gal4/+ and *UAS-THmiR-G*/+ (figure 5*a*; two-way ANOVA results in table 1; effect of isolation: *F*_1,42_ = 19.13, *p* < 0.0001; genotype and isolation interaction: *F*_2,35_ = 8.635, *p* = 0.0009). In females, we observed a decrease in the number of flies within four body lengths for all three genotypes after isolation and a decrease in *TH>THmiR*-G flies compared with their genetic controls (figure 5*b*; two-way ANOVA results in table 1; effect of genotype: *F*_2,37_ = 4.009, *p* = 0.0265; effect of isolation: *F*_1,37_ = 21.91, *p* < 0.0001).

When investigating the recovery, we observed no difference in any genotype between group-housed and recovery treatments in males (figure 4*c*; two-way ANOVA results in table 1) or females (no genotype was different comparing group housed to recovery treatments—figure 5*d*; two-way ANOVA results in table 1; effect of genotype: *F*_2,35_ = 5.124, *p* = 0.0112).

To establish the importance of DA in a response to isolation and recovery, we performed LC/MS on the heads of males and females to quantify changes in DA levels after isolation and recovery. After isolation, males had decreased DA levels, whereas females’ DA levels remained similar to those of group-housed flies. Furthermore, male DA levels returned to a similar level as group housed after a recovery period. Females’ DA levels remained unchanged (figure 5*e*; two-way ANOVA results in table 1; effect of social experience: *F*_3,27_ = 3.043, *p* = 0.0459).

Taken together, these results indicate that DA is important for social space in response to isolation and recovery in males but not females and that DA levels are influenced by previous social experience in a sex-specific manner.

### 3.7. *nlg3^Def1^* flies have less dopamine, but its levels are unchanged after isolation

**Figure 6.**
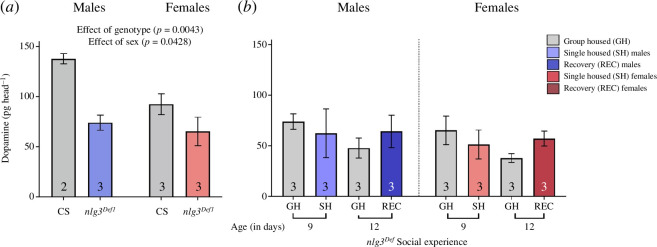
*nlg3^Def1^* flies have less DA, and those levels are not altered by social experience. (*a*) DA levels (pg head^−1^) in CS and *nlg3^Def1^* males (blue shade) and females (red shade). *nlg3^Def1^* flies had less DA when group-housed and isolated compared with group-housed CS, with females showing a lesser amount overall (two-way ANOVA—effect of genotype: *F*_1,7_ = 17.21, *p* = 0.0043 and effect of sex: *F*_1,7_ = 6.107, *p* = 0.0428). (*b*) DA levels (pg head^−1^) in male (blue shade) and female (red shade) group-housed and recovery *nlg3^Def1^* flies are unchanged with social experience. Appropriate age comparisons were made (refer §2 for details). Bars: mean ± s.e.m. Grey shades: group housed (GH); coloured shades: social isolation (SH) or recovery from social isolation (REC), as indicated; blue shades: males; red shades: females. On the graph, the results of the post hoc tests are shown only when *p* < 0.05. The results of all the comparisons, significant or not, can be found in tables 1–3. *n* = 3–6 for all treatments. GH: group housed; SH: single housed; REC: recovery from social isolation.

To determine whether an interaction was occurring between DA and *nlg3,* we tested DA levels in group-housed CS and *nlg3^Def1^.* In both males and females, DA was decreased in *nlg3^Def1^* compared with CS, and females had lower DA levels compared with males (figure 6*a*; two-way ANOVA results in table 1; effect of sex: *F*_1,7_ = 6.107, *p* = 0.0428; effect of genotype: *F*_1,7_ = 17.21, *p* = 0.0043). However, when we tested the effect of social experience, no difference in DA levels was observed between *nlg3^Def1^* group housed, isolated and recovery males and females (figure 6*b*; two-way ANOVA results in table 1).

## 4. Discussion

Here, we report the effects of social isolation on social space and sociability. In addition, for the first time to our knowledge, we report the effect of social recovery on the fly social behaviour. Social space was increased, and sociability decreased after isolation but was recovered after 3 days of group housing following isolation. We also show that mating status affected the response to isolation in a sex-specific manner and that as soon as two flies or more are present, the size of the group prior to assaying social space has no effect on social spacing. In addition, we show that an autism candidate gene, *nlg3,* important for the response to social experience, is not required for recovery from social isolation to occur. We also further confirm the role of DA in social space. However, we show for the first time to our knowledge, the sex-specific role of DA in social spacing: DA is not only required for a response to isolation but also for recovery from isolation in males, but not females. Furthermore, DA levels also respond to the social environment by decreasing in males after isolation and returning to normal levels when recovery occurs, but not in females. Finally, we show that a *nlg3* loss of function mutant, *nlg3^Def1^*, inhibits a change in DA in males and females, with no further changes added with social isolation.

We first looked at how long flies needed to be isolated to effect social space. We saw a sex-specific response to isolation where males only needed 2 days of isolation while females needed closer to 7 days. The females were less effected in the early days of isolation and seemed to be more resilient to the effects of isolation than males (figure 2*a*,*b*). Our results for both males and females isolated for 7 days phenocopy the results of Simon *et al*. [41]. With regard to recovery, males also responded faster and recovered in 2 days versus 3 days in females (figure 2*d,e*). In summary, although males were affected sooner by isolation, they were also able to recover faster than females. The males’ quick response to their social environment may be driven by their need to be quickly adaptable and ready for the possibility of sperm competition, which is affected by perception of a rival [80,81]. Finally, we showed that sociability is reduced in males and females but recovers after group housing (figure 2*c*,*f*), indicating that isolation is affecting multiple social behaviours in the flies. Similarly, for males, increasing isolation length from 1 to 4 days leads to increased effect on aggression [36].

As we have previously shown that mating status and isolation are important for social space [41,42], we investigated the role of mating status in social space in response to isolation. In both males and females, social space was increased in virgin group-housed flies compared with mated group-housed flies as previously reported ([41]; figure 3*a,b*). Both virgin and mated male flies had increased social space after isolation. We then wanted to know whether the number of flies present while group-housed affected social space. We tested single-housed flies and flies reared group housed with 2, 6, 16 and an uncontrolled random number of flies. The isolated flies had increased social space as expected (figure 3*c,d*). Group-housed flies had lower social space than isolated flies; however, the social space was similar among all the densities indicating the presence of even one other fly is enough to avoid the negative consequences of isolation on social space. Similarly, the presence of another fly is also sufficient to almost eliminate the effects of isolation on aggression in flies [36].

Next, we looked at social space in the *Drosophila* homologue of an autism candidate gene, *nlg3*, after isolation and recovery. We showed that males CS and *nlg3^Def1^* flies had increased social space after isolation. We have previously reported a diminished response to isolation in *nlg3^Def1^* flies [42]; however, this time, we observed that males responded to isolation similarly to their genetic controls (figure 4*a*). However, isolated *nlg3^Def1^* females did not, similar to what we had reported previously (figure 3*b*; [42]). When testing the recovery after isolation, male and female CS flies had similar social space to flies always group housed, indicating recovery had occurred (figure 3*c,d*). Interestingly, in *nlg3^Def1^* flies, both males and females had decreased social space in the recovery treatment, which appears as an over compensatory response. In an independent repeat of those data, both CS and *nlg3^Def1^* flies recovered similarly from isolation (electronic supplementary material, figure S3). In both cases, the lack of *nlg3* did not prevent the recovery from social isolation.

Using an RNAi against *TH*, we were able to show that DA is important for a response to isolation in males since the isolated flies did not respond to isolation and even had a slightly closer social space in isolated compared with group-housed flies (figure 5*a,*c). A similar result was reported by Xie *et al.* [43], where group-housed flies acted as if isolated and isolated flies acted as if group housed. We do not see as strong of effect as those authors reported, but we also used a pan-TH driver, while Xie *et al.* [43] focused on a few TH-expressing cells only. Our work and that of Xie *et al.* [43] demonstrate the importance of DA in males in the response to the social environment. However, we show for the first time to our knowledge, that females do not require DA for a response to isolation and recovery to occur (figure 5*b,d*). Another neurotransmitter or neuromodulator could be at play in females, in response to social experience. It has been previously shown that both males and females respond similarly to reduced DA levels, when group housed, with an increase in social space [48]. However, DA might not be the neurotransmitter involved in responding to social experience in females.

When we isolated our control line, CS, we confirmed that there is a decrease in DA in males but not in females and that the DA levels in males returned to the level of group-housed flies after recovery (figure 5*e*). Ganguly-Fitzgerald *et al.* [31] also saw a decrease in DA in males after isolation. Taken together, these results indicate that DA in males is extremely important for social space in response to isolation, but not in females. Finally, we report that DA levels in *nlg3^Def1^* remain unchanged after isolation, providing further evidence that *nlg3* and DA are both contributing to the modulation of social space after isolation (figure 6*a,b*). In both CS and *nlg3^Def1^,* decreases in DA were observed between 9- and 12-day-old flies regardless of social experience, consistent with age-related decreases in DA reported previously [82]. By contrast, the social space of group-housed CS flies has not been reported to change before they reach 14 days of age [46,47]; and those ages of 12- and 14- days-old might even be quite advanced, for a fly in its natural habitat. Indeed, *D. melanogaster* typically have a lifespan averaging only 6 days [83], considerably shorter than their lifespan in captivity (up to three months—for review see [84]; which is why we decided to limit our study to periods of isolation of no more than 7 days).

**Figure 7.**
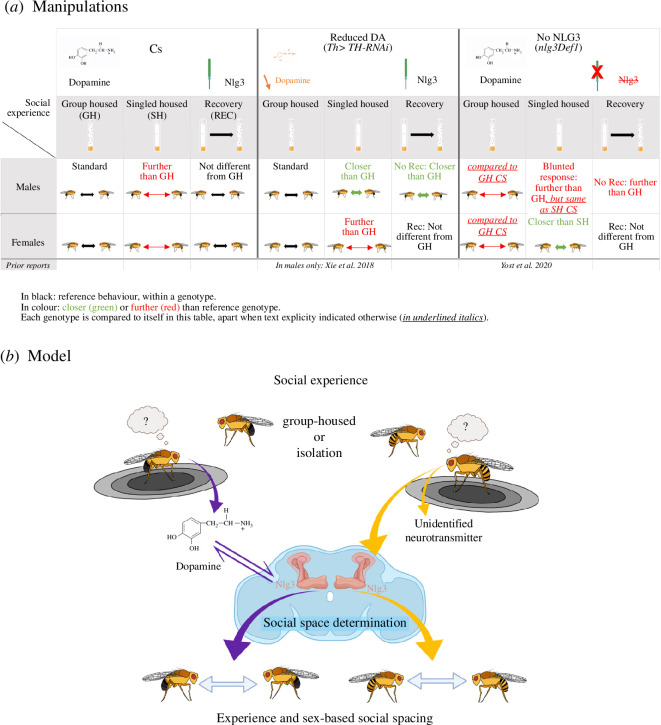
(*a*) Manipulations performed in this study. (*b*) Working model: *nlg3* and DA are part of a pathway responsible for the modulation of social behaviour after isolation in males. *nlg3* would be required downstream of DA, for proper DA signalling in response to the social environment, with some feedback regulation. Females do require *nlg3* for a typical response to isolation, but other neurotransmitters or neuromodulators, responsive to social experience in females, may be interacting with *nlg3*. Blue outline: *Drosophila* brain outline. Created with BioRender.

In the past decade, *Drosophila* has emerged as a model to study the underlying mechanisms of neuropsychiatric disorders [85–94], despite its limitations [84]. In that context, what does recovering from social isolation mean for DA and an autism-related gene? DA in males is required for a response to the environment; however, the flies need *nlg3* for DA levels to change in response to the social environment and subsequently modulate behaviour. Furthermore, DA levels decrease in response to social isolation in males, but the NLG3 protein itself is not responding to social experience [42]. We predict that *nlg3* and DA are part of a pathway responsible for the modulation of social behaviour after isolation in males. *nlg3* would be required downstream of DA, for proper DA signalling in response to the social environment, with some feedback regulation since DA did not change after isolation when *nlg3* was absent. Females do require *nlg3* for a typical response to isolation [42], but other neurotransmitters or neuromodulators, responsive to social experience in females, may be interacting with *nlg3* (figure 7). For example, *nlg3* has been shown to affect glutamate receptor GluRIIA recruitment at the larval neuromuscular junction [95]; however, the involvement of glutamate in social spacing seems limited, at least in males, and not reported for females [64]. Similarly, neuromodulators of social space in flies only studied in males, such as acetylcholine [64]), while others have been studied in both males and females such as serotonin [63,65] and GABA [62]. These neurotransmitters should further be investigated for their role in a response to social isolation in females. Also, whether other genes, neurotransmitters and neuropeptides involved in the response to social experience, in both males and females, such as those recently reviewed by Yadav *et al.* [20], also modulate changes in social space and sociability is still unknown.

A role of Nlgs in DA signalling, and their common influence on behaviour, including social behaviour, has been observed in mice [77,96], the northern swordtail fish [97] and the worm *Caenorhabditis elegans* [75]. What remains unknown is whether *nlg3* is directly involved in the post-synaptic recruitment of one of the DA receptors in flies, as has been suggested, based on work in mice, by Uchigashima *et al.* [76].

Beyond *nlg3* and DA, other Nlgs, synaptic proteins and neuroreceptors have been shown to influence proper social spacing. As mentioned earlier, *nlg2* and *nlg4* [68], acetylcholine [64] (all of those only studied in males) and GABA [62] are intriguing candidates. Indeed, *nlg4*, which is important for social spacing, also modulates GABA neurotransmission, although this modulation was studied in the context of sleep regulation. However, GABA is crucial for normal social spacing, as demonstrated by the consequence of its mislocalization in mitochondria, in CYFIP haploinsufficient flies [62].

Of note, sexual dimorphism in social spacing in flies has also been reported in mutation of another candidate gene for neuropsychiatric disorders. Indeed, the effect of downregulating the expression of *dABCA (ATP-binding cassette protein A)* was stronger in males than in females [54]. A link with sexual dimorphism in the underlying neurocircuitry itself has been found in *C. elegans*, where Neuroligin post-synaptic binding partner, Neurexin, is localized in and affects the function of a sexually dimorphic neuron (which happens to be GABAergic [98]). And the DA-signalling circuitry is known to be sexually dimorphic, at least in its response to stress [99]. Although, unlike in humans, those examples are unrelated to sex chromosomes, they might inform the molecular basis and neural circuitry of sex differences observed in the severity and occurrences of disorders such as autism spectrum disorders [100].

Future research in the field will need to combine these various data to offer an integrated and more comprehensive understanding of how the players underlying social spacing behaviour work together. For example, there might be a structural association between CYF1P and Nlgs, as both affect the actin cytoskeleton [56,101–104].

In conclusion, for the first time to our knowledge, we have demonstrated that social space and sociability are recoverable after isolation in a sex-specific manner. We have also shown that DA is required for the recovery from social isolation in a sex-specific manner and that *nlg3* is required for DA levels to respond to the social environment. If those gene by environment by sex interactions are evolutionarily conserved, it might help us better understand the molecular basis of the social difficulties encountered by humans with neurodevelopmental disorders in response to changing social environments.

## Data Availability

The datasets analysed for this study can be found in the Dryad Digital Repository [105]. Supplementary material is also available online [106].
